# Tissue palpation in endoscopy using EIT and soft actuators

**DOI:** 10.3389/frobt.2024.1372936

**Published:** 2024-08-09

**Authors:** Amirhosein Alian, James Avery, George Mylonas

**Affiliations:** The Hamlyn Centre, Imperial College London, London, United Kingdom

**Keywords:** soft robotics, tactile sensing, palpation, medical robotics, endoscopy, artificial intelligence

## Abstract

The integration of soft robots in medical procedures has significantly improved diagnostic and therapeutic interventions, addressing safety concerns and enhancing surgeon dexterity. In conjunction with artificial intelligence, these soft robots hold the potential to expedite autonomous interventions, such as tissue palpation for cancer detection. While cameras are prevalent in surgical instruments, situations with obscured views necessitate palpation. This proof-of-concept study investigates the effectiveness of using a soft robot integrated with Electrical Impedance Tomography (EIT) capabilities for tissue palpation in simulated *in vivo* inspection of the large intestine. The approach involves classifying tissue samples of varying thickness into healthy and cancerous tissues using the shape changes induced on a hydraulically-driven soft continuum robot during palpation. Shape changes of the robot are mapped using EIT, providing arrays of impedance measurements. Following the fabrication of an in-plane bending soft manipulator, the preliminary tissue phantom design is detailed. The phantom, representing the descending colon wall, considers induced stiffness by surrounding tissues based on a mass-spring model. The shape changes of the manipulator, resulting from interactions with tissues of different stiffness, are measured, and EIT measurements are fed into a Long Short-Term Memory (LSTM) classifier. Train and test datasets are collected as temporal sequences of data from a single training phantom and two test phantoms, namely, A and B, possessing distinctive thickness patterns. The collected dataset from phantom B, which differs in stiffness distribution, remains unseen to the network, thus posing challenges to the classifier. The classifier and proposed method achieve an accuracy of 93% and 88.1% on phantom A and B, respectively. Classification results are presented through confusion matrices and heat maps, visualising the accuracy of the algorithm and corresponding classified tissues.

## 1 Introduction

Robotic technologies play a significant role in healthcare, contributing to both diagnostic and therapeutic interventions Avgousti et al. (2016). This level of attention stems from their ability to enhance surgical capabilities through improved dexterity, precision, and robustness. The introduction of surgical robotics has revolutionized Minimally Invasive Surgeries (MIS) Advincula and Wang (2009), transforming traditional laparoscopy approaches Williamson and Song (2022). However, conventional surgical robots, characterized by rigid and high-stiffness components, pose challenges in ensuring safe interaction with the patient’s body. Concerns arise, particularly in high-force operations, where the risk of ruptures and perforations becomes a notable issue. To address the concern, soft robotics emerges as a viable alternative to the field. Leveraging concepts that have found success in various technological fields over the past two decades, soft robotics offers extended compliance, hyperelastic behaviour, and characteristics resembling human tissues Cianchetti et al. (2013); Rateni et al. (2015); Cianchetti et al. (2014).

Soft robotics demonstrate noteworthy capabilities in enhancing maneuverability, ensuring safety, and adapting to diverse environments, rendering them effective assets in surgical interventions. These attributes contribute to the realization of “theranostics,” encompassing both diagnostic and therapeutic treatments Kwok et al. (2022). The prominence of soft robotics is evident in numerous medical applications, including peripheral lung diagnostics and interventions Nguyen et al. (2023), tissue retraction Amadeo et al. (2022), breast cancer detection Berthet-Rayne et al. (2021), and tissue sampling Van Lewen et al. (2023). Similarly, the advantages of soft robotics extend to lower gastrointestinal endoscopy, where high flexibility is required to negotiate tortuous paths while minimizing postoperative complications Chauhan et al. (2021). Explorations into MIS applications have involved soft robots designed with continuum mechanisms, introducing novel locomotion methods for inspecting the gastrointestinal tract Tolley et al. (2014); Stilli et al. (2014). Additionally, their effectiveness in delivering therapeutic interventions is validated by the employment of resilient yet flexible designs capable of exerting high forces Runciman et al. (2020).

Many soft endoscopes require a clear and unobstructed line of sight, typically facilitated by an endoscopic camera or alternative intraoperative imaging Fu et al. (2021), to navigate the endoscope or visualise lesions. However, in colonoscopy, visual-oriented diagnosis faces challenges in early-stage cancer detection, requiring soft tactile sensors to diagnose non-polypoid lesions Wang et al. (2016). Conversely, early malignant tissues exhibit distinctive mechanical properties, such as stiffness, differing from healthy tissues. This disparity highlights the relevance of tactile sensing for tissue palpation Stewart et al. (2018); Ahn et al. (2010); Lopez et al. (2011). Table 1 summarizes the conventional and state-of-the-art tactile sensors in MIS, categorized by their technological designs Othman et al. (2022). These sensors can be implemented by making indentations in tissues, catheterization, and non-contact approaches Konstantinova et al. (2014).Utilizing touch for tissue manipulation enhances identification of lesions Scimeca et al. (2022), achievable by soft tactile sensors with human-level accuracy Gwilliam et al. (2010) employed to detect the depth of hard inclusions within soft tissue Ahn et al. (2012); Nichols and Okamura (2013); Zhang Qiu et al. (2022); Yan and Pan (2021). Conventional robot-assisted palpation, often with rigid manipulators, relies on force measurements during *ex vivo* operations, requiring tissue removal Xiao et al. (2020). Another category of tactile sensors integrates with laparoscopic or endoscopic instruments, providing intra-operative tactile diagnosis without tissue removal Tanaka et al. (2010). Some use optical fibres to sense tissue stiffness Wanninayake et al. (2012); Lv et al. (2020); Tang et al. (2021), offering high resolution but increasing cost and complexity Othman et al. (2022). Additional tactile sensors measure the force exerted by miniaturized and articulated probes, providing information about tissue properties Sornkarn and Nanayakkara (2017). Chuang *et al.* developed a piezoelectric sensor for colonoscopies, mounted at the colonoscope tip, capable of detecting tissue stiffness variations Chuang et al. (2016). Fluid-driven tactile sensors, enabling cost-effective and easy fabrication, identify tissue stiffness through volume changes Zhao et al. (2023b) or fluidic channel impedance variations Avery et al. (2020). The sensors outlined in the literature either require tissue excision or depend on separate devices for mounting and operation.

**TABLE 1:** A summary of the conventional tactile sensors in MIS.

Sensing technology	Advantages	Disadvantages	References
Piezoresistive/ Piezoelectric	- Low cost - High sensitivity - Repeatable	- Prone to hysteresis - Accuracy loss in long-term	Ahmadi et al. (2010) Radó et al. (2018) Sornkarn and Nanayakkara (2017)
Capacitive	- Ease of design - Thermal noise immunity - Tunable resolution	- Prone to hysteresis - Non-linear	Lee et al. (2015) Kim et al. (2018)
Optical	- Versatility - Miniaturization - Lightweight - MRI compatible	- Complex design - High cost	Ahmadi et al. (2011) Polygerinos et al. (2010) Lv et al. (2020) Tang et al. (2021)
Microfluidic/ Fluidic	- High flexibility - Low-cost - Easy fabrication	- Leakage - Limited stability in long-term	Zhao et al. (2023a) Avery et al. (2020) Kara et al. (2023)
Imaging-Based	- High resolution - Large area coverage - Minimized coverage	- Large-sized - Restricted camera focal distance	Venkatayogi et al. (2022) Trueeb et al. (2020) Kawahara et al. (2010)

**FIGURE 1:**
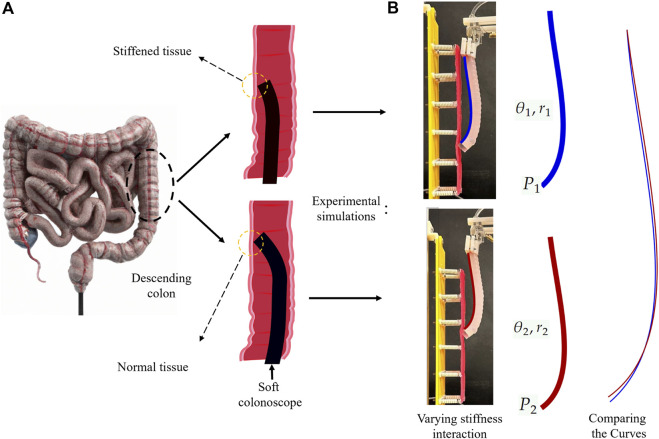
The schematics and experimental simulations of the proposed approach, which is based on the shape changes resulted from the interaction with different stiffness of tissue. **(A)** schematic illustration of a soft colonoscope sliding against the descending colon tissue. **(B)** Replicating the schematic representation in experimental simulations. Different shape configurations (e.g., bending angle and radius) over interaction with varying stiffness tissue samples.

This paper proposes an Electrical Impedance Tomography (EIT)-based tactile sensing scheme embedded in a soft manipulator suitable for colon tract navigation. The method enhances hydraulically-driven soft endoscopes with proprioceptive tactile sensing for tissue palpation, without any additional modalities, aiming to improve their early-stage cancer diagnostic capabilities. The proposed method minimizes system complexity and integration challenges while enhancing dexterity in endoscopes with multiple degrees of freedom Treratanakulchai et al. (2022). The sensor operates by measuring varied deformation of the continuum endoscope during interaction with tissues of different stiffness, complementing the concurrent detection of visible polyps through the endoscopic camera. The interaction between a flexible continuum robot and the environment alters system stiffness, reflecting external force and displacement at the contact point, corresponding to the elasticity of the target Stella et al. (2023). Thus, the configuration of the robot in a static equilibrium depends upon the stiffness of the contact tissue. In oncological diagnostics, early-stage malignant colon tissues exhibit increased stiffness compared to benign counterparts Stewart et al. (2018). Conventional approaches relying on cameras may overlook these stiffened tissues. In a hypothetical scenario, if a suitably soft endoscope probes the colon tissue while negotiating the lumen, the tissue stiffness can be manifested in the backbone shape of the endoscope as varying bending curves (see Figure 1A). Tactile feedback provided by shape information, coupled with visual information, enhances the detection of elusive cancerous tissues. The proposed scheme measures electrical impedance variations induced by shape deformation along the endoscope’s working channel. Saline serves as the conductive fluid for mapping impedance measurements through nine channels. Machine learning models analyze these impedances, facilitating the classification of early-stage malignant tissues. To validate the approach, a suspended tissue phantom representing the colon and mesentery is designed. The phantom incorporates silicon tissue samples with varying thicknesses, mimicking stiffness changes along the colon. The soft continuum robot traverses vertically, recording sequences of data, including pressure and impedance values, upon interaction with the tissue phantom.

## 2 Materials and methods

To demonstrate tissue palpation using soft continuum endoscopes, we explored the proposed sensing capability using an elastomer bending manipulator with integrated EIT sensing, capable of in-plane deformation. This hydraulically-driven manipulator represents typical hyperelastic soft continuum robots used as soft endoscopes in the literature Manfredi et al. (2019); Zhang et al. (2019); Abidi et al. (2018). EIT, previously developed for deformation measurements and tactile force predictions in soft actuators Alian et al. (2023), serves as the core sensing modality. The soft manipulator simulates navigating the descending colon for demonstration and procedural simulation. A tissue phantom, resembling colon tissue suspension and the mesentery, is fabricated for this purpose. The continuum manipulator palpates the tissue phantom while a vertical linear motor lifts it along the simulated colon. Collected sensory data, including pressure and EIT values, are interpreted and classified using a Recurrent Neural Network.

### 2.1 Soft continuum manipulator fabrication and EIT integration

**FIGURE 2:**
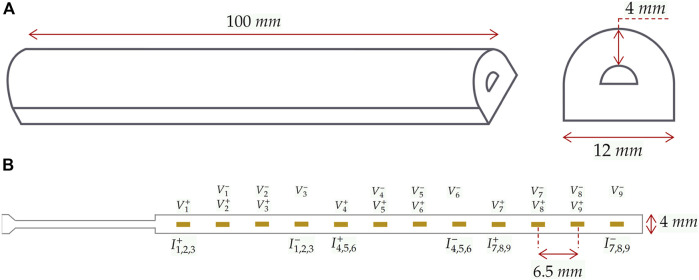
Soft continuum robot and integrated electrodes. **(A)** dimensions of the manipulator and its cross-section. **(B)** The specifications of the FPC and the assignment of electrodes to measurement and injection pairs in the selected protocol. Measurement and injection electrodes are denoted by V,I , and positive and negative poles are denoted by +, −, respectively. The indexing of each electrode refers to the corresponding channel. For instance, $I_{1,2,3}^{+}$ under the first electrode demonstrates its role as the injection positive pole in channels 1, 2, 3.

The manipulator, Figure 2A, following the procedure outlined in Polygerinos et al. (2015), is constructed using a three-component mold filled with Ecoflex 00–50 Silicone. With a length and diameter of 100 mm and 12 mm, respectively, and a wall thickness of approximately 4 mm, it forms a semi-circle inner chamber with 4 mm width. The inner chamber is hydraulically inflated and produces in-plane unidirectional bending motions towards the tissue phantom. Nylon fibres and an inextensible polyester layer are incorporated to restrict radial expansion and minimize longitudinal tension.

Integrating EIT requires an array of electrodes, an impedance spectroscope, and a conductive fluidic medium. The impedance spectroscope stimulates electrical current and measures corresponding voltages, reflecting changes in the conductivity of the medium. Stimulation and measurements follow a protocol determining which electrodes are used for current injection and impedance measurements. In the experiments, a 9-channel EIT protocol is applied, utilizing a linear array of 12 electrodes on a Flexible Printed Circuit (FPC) made of a Polyamide film. Each channel includes a pair of injection electrodes and a pair of measurement electrodes. The schematics of the protocol and formation of the electrodes are illustrated in Figure 2B, where $I_{i}^{+}$ and $I_{i}^{-},i=\left[1\;9\right]$ denote the positive and negative poles of injection pairs for the *ith* channel, respectively. Positive poles of measurement pairs are represented as $V_{i}^{+}$ , whereas $V_{i}^{-}$ represents negative measurement poles. For instance, $I_{1,2,3}^{+}$ under the first electrode in Figure 2B denotes its positive injection pole in channels 1, 2, 3. The electrodes can act as both positive and negative poles but in different channels, depending on the protocol.

The integration of EIT into the soft manipulator involves embedding and fixation of the FPC into the inner chamber of the manipulator. Assuming the manipulator undergoes pure bending, the manipulator’s inner chamber geometry defines the flat side as the neutral surface, suitable for non-stretchable FPC placement. Upon pressuring the manipulator using 0.9% saline as the conductive medium, the varying impedance measurements collected by the spectroscope are observed. The deformations upon bending increase the cross-section of the inner chamber due to the restricted elongation. These changes are associated with the pressure values and affect the impedance readings according to Pouillet’s law. The increased area leads to the reduced impedance values, which subsequently are alluded to the backbone shape changes of the manipulator. EIT data are recorded using the Quadra impedance spectroscopy developed by Eliko tech Min et al. (2018). The device incorporates an alternating current source capable of generating signals with 15 frequencies up to 349 kHz. Additionally, the spectroscope records the data using a data acquisition unit and a multiplexer at varying frame rates based on the number of channels in the protocol. Herein, the 9-channel protocol is sampled at 20 Hz by injecting a current signal of 1 mA amplitude. The reference data are collected using the current signal at the frequency of 61 kHz, sampled through Time Division Multiplexing (TDM).

### 2.2 Colon tissue phantom simulator

**FIGURE 3:**
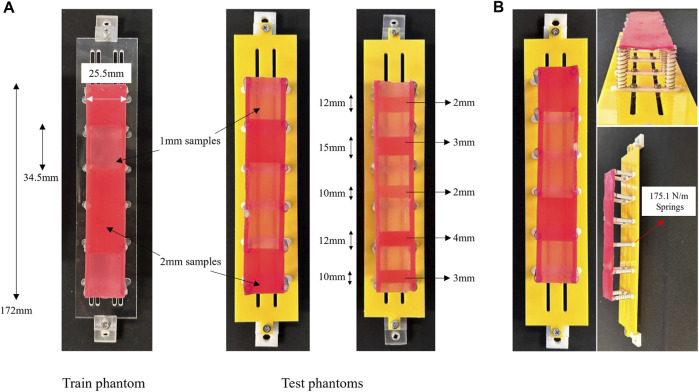
**(A)** The phantoms used for training and test dataset collection together with their specifications. **(B)** The fabricated tissue phantom simulator based on a mass-spring model and composed of Silicone samples of varying stiffness.

To replicate colon tissue stiffness, a set of phantoms are designed, considering the colon tissue suspension. Typically, phantoms overlook the normal stiffness of colon tissue, essential to mimic the resistance of ligaments and surrounding organs. The proposed phantoms in this study enable tactile sensory data collection. These phantoms, categorised into training and test phantoms, follow a mass-spring model. As shown in Figure 3B, an artificial tissue segment is suspended to a rigid component via compression springs with a constant of 175.1N/m Under quasi-static loading and a fixed spring constant, the elasticity modulus of 0.425 MPa is computed using the spring geometry (25 mm length, 6.5 and 1 mm outer and wire diameters, respectively), aligning with Massalou et al. (2019) results in the circumferential direction. The springs model the resistance yielded by ligaments and neighbouring tissues upon the palpation of the colon wall.

The tissue segment consists of Ecoflex 00–50 (Smooth-On, Inc., Macungie, PA, United States) silicone cast into 3D printed moulds, providing a location-based stiffness varying pattern through thickness variations. Stiffness variations are subsequently categorised through a binary classifier. The training phantom includes five tissue samples of 1mm and 2 mm thickness, with the latter indicating unhealthy tissues. The samples, measuring 34.5 mm in length and 25.5 mm in width, are randomly distributed across the segment. In Figure 3B, thicker tissue samples are identified by a darker colour. Test phantoms, A and B, follow similar fabrication principles, but A has an alternative distribution pattern of 1 mm and 2 mm samples, and B is composed of two additional samples of 3 mm and 4 mm thicknesses. While the lengths of the samples on phantom A are identical, they differ on B as depicted in Figure 3A. The varying design factors present in phantom B, namely, sample length and thickness, serve as a means to investigate the generalization of the proposed classifier. These tissue phantoms, combined with the soft manipulator, are employed to simulate *in vivo* colon palpation. A data-driven classification model will interpret tactile data collected during the procedure.

## 3 Experimental setup

Tissue palpation involves probing the tissue while traversing the surface for data sampling at various locations. Two classes of actuation units are employed: one for probing motion and the other for manipulator displacement. The number of units for each class depends on the required Degrees of Freedom (DOF) for each motion. Here, the 2D plane is targeted with 1-DOF probing motion and vertical line traversal, requiring one actuation unit per motion. Hydraulically pressurising the manipulator with a syringe pump system, driven by a linear stepper motor, generates in-plane probing motion. Stepper motors, controlled by S-lite and S series uStepper drivers, with a resolution of 400 steps per revolution, actuate the traversing and probing motions, respectively. Vertical manipulator displacements are recorded using a built-in encoder.

To probe the tissue, the manipulator tip indents the phantom, ensuring the exerted normal force stays within a safe range. The phantom is vertically mounted on an aluminium structure, and the manipulator is attached to the moving stage of a vertical linear motor, moving parallel to the phantom. Prior to initializing the experiments, the manipulator faces the tissue phantom with its flat side parallel to the phantom surface. To replicate *in vivo* trials, the manipulator’s rest position is set within colon dimensions, ensuring its relative position to the phantom aligns with values from Finocchiaro et al. (2023). In Figure 5 at t=0 , the manipulator is configured at its rest position with an absolute pressure of 1.260bar , recorded using an MS5803-14BA sensor with a resolution of 1mbar . Pressure data were recorded throughout the experiments, transmitted to an Arduino Due at 20 Hz through an I2C interface.

### 3.1 Data collection

**FIGURE 4:**
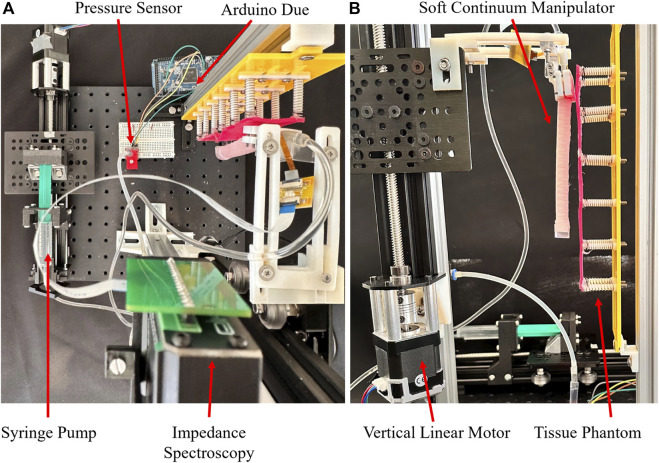
The experimental setup consisting of the tissue simulator, actuation units, continuum manipulator, and sensing devices. **(A)** top view, **(B)** front view.

**FIGURE 5:**
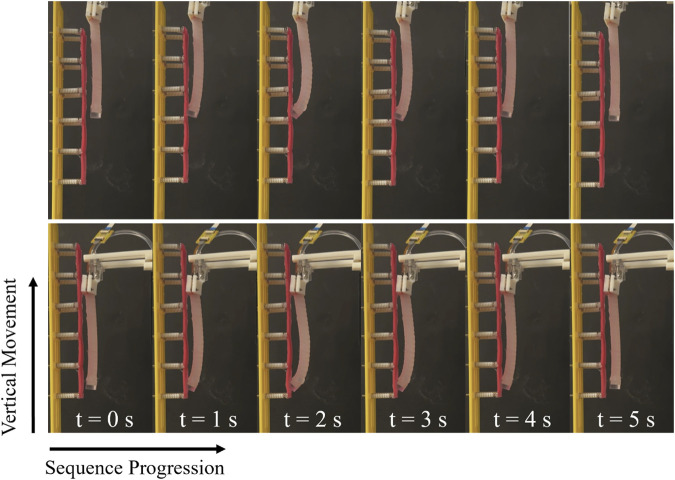
The inflation and deflation of the manipulator during data sampling, and the direction of traversing.

Two series of experiments were conducted. The first illustrates EIT output distinctions while probing tissue samples of varying thicknesses on the training phantom (Figure 3A). The second involves joint data collection on the training and test phantoms, followed by implementing a data-driven classifier. The experimental setup and required components for actuation and data acquisition are shown in Figure 4. In the first experiment, pressure is applied to the manipulator in trapezoid signal waves with a constant high value of 1.750bar and a high time of 2 s. The pressure is produced by the stepper motor rotating at the maximum speed of 2000steps/s and acceleration of $6000\;steps/s^{2}$ . This process is repeated at five different vertical positions of the manipulator tip, corresponding to distinctive parts of tissue samples with 1 mm and 2 mm thicknesses. These locations include the top, middle, and bottom sections of each sample. The experiment was repeated 10 times to assess the repeatability and separability of the EIT signals. In practical scenarios, factors such as the distance between the tip and tissue surface or the initial manipulator pose can vary randomly. Uncertainties involved during palpation due to the presence of complex colon anatomy and undesired motion of the manipulator render the surgical site unpredictable. This heightened uncertainty challenges the differentiation of tissue stiffness based on EIT outputs. To observe the impact of these uncertainties and create a more generalized dataset, the manipulator is pressurised with randomly bounded inputs from 1.25bar to peak values ranging from 1.6 to 1.8bar . Despite the added complexity by randomising the pressure inputs, the overall robustness of the network is enhanced. Triangular wave signals of pressure (depicted in Figure 7) inflate the manipulator instead of trapezoid ones. The bounds (1.6 and 1.8bar ) ensure a safe interaction between the manipulator and the phantom while keeping the exerted force within a permissible threshold. The dataset from the second experiment comprises both training and testing data. The training data are acquired as the manipulator traverses and probes the training phantom (Figure 5). Similarly, the test data come from palpating test phantoms A and B. Both training and testing data collection started with the manipulator positioned at the bottom of the phantom and gradually moved to the top. The manipulator traverses the phantom at a rate of 2 mm every 3 min during training, while the test data collection rate is set at 1 mm every 12 s.

### 3.2 Data post-processing

The data required for classifying palpated tissues include pressure values, EIT outputs, and the vertical displacement of the manipulator. Collected pressure and displacement datasets from Arduino and uStepper boards, respectively, are communicated to MATLAB 2022. These datasets, stored as string arrays with corresponding timestamps, are processed further. Impedance values, sampled at 20 Hz, are logged as text files and formatted into string and numerical arrays in post-processing. Pressure and EIT temporal sequences will be used as classifier inputs, while displacement data are used for labeling and illustration only. Each input sequence represents a single cycle of manipulator inflation and deflation with corresponding EIT changes. Despite nominal similar sampling rates for pressure and EIT data, variations in start and end points necessitate data synchronization. Piecewise Cubic Hermite Interpolating Polynomial (PCHIP) interpolation ensures consistent data points in each sequence, maintaining the same number of pressure and EIT measurements. Finally, the data are organized into train and test datasets, creating an *n-by-d* matrix for each sequence, where *n* represents the feature space size and *d* denotes the number of data points. The feature space size is ten (pressure and 9-channel EIT values) for all sequences, with *d* varying among them.

### 3.3 Machine learning model

Due to the intrinsic nonlinear and hyperelastic nature of the continuum manipulator and tissue phantom, linear separability of data featuring different stiffness levels is not feasible. Colon tissue, in particular, exhibits isotropic and anisotropic hyperelastic biomechanics with time-dependent behaviour Bhattarai et al. (2021). Thus, recognizing nonlinear and temporal patterns in the data requires data-driven models for classifying colon tissue stiffness into healthy and cancerous categories. In the literature, Convolutional and Deep Neural Networks (CNN, DNN) are common to classify colorectal cancer images utilizing Visual Geometry Group networks (VGGs) Anju and Vimala (2023) and Residual networks, also known as ResNets Sarwinda et al. (2021). The overall structure of VGGs includes convolutional layers followed by fully connected layers Ferreira et al. (2018). ResNets are deep architectures used to reduce training time. They establish shortcuts by skipping connections between specific layers, addressing the issue of vanishing gradients in convolutional neural networks.

Recurrent Neural Networks (RNN) are well-suited candidates for time-dependent data Yu et al. (2019), incorporating feedback loop connections to handle sequential input data of varying lengths. However, RNNs face challenges like vanishing and exploding problems due to their limited memory capacity. Alternatively, Long Short-Term Memory (LSTM) networks, as an improved class of RNNs, have been introduced Gers et al. (2000) with additional gates to the standard recurrent cell. This amendment improves the remembering capacity and addresses the aforementioned issues in RNNs. Given the variable sampling rate of the sensing modalities of the setup, resulting in varying-size data sequences, the inherent ability of LSTMs to handle this type of data renders them suitable.

In this study, a 6-layer LSTM network serves as the core algorithm for classifying the dataset into healthy and unhealthy tissues. The network comprises an LSTM layer, a drop-out layer, and a softmax layer. Training data consists of 1,057 sequences, reducing to 427 sequences for the test datasets on phantoms A and B. The input size, representing the feature space dimension, consists of nine vectors for EIT channels and one pressure value. With a 2-sized output denoting healthy and unhealthy tissues, the network is configured accordingly.

Prior to inputting data into the classifier, the dataset is normalized and sorted based on sequence length to minimize the padding extent added by the network, thereby optimizing the mini-batch size. The feature normalization is performed using the maximum and minimum values of the datasets, resulting in scaling the feature variables to a range from 0 to 1. The LSTM classifier is trained using Adam solver and the execution environment is set to automatic. Adam enables faster convergence of the parameters by dynamically adapting the learning rate. The solver is suitable for noisy input data and robust to hyperparameter tuning. Alternatively, Nesterov-accelerated Adaptive Moment Estimation (NAdams) can be used for faster and more generalized convergence. Training and validation of the network are implemented in MATLAB R2022a and the learning algorithm iterated over 500 epochs. The hyperparameters of the network including the Number of Hidden Units (NHU), Initial Learn Rate (ILR), and Mini-Batch Size (MBS) are tuned through the Bayesian optimization algorithm Bull (2011), within the corresponding ranges of [1,100] [0.0001 0.1], and [1,300]. After 100 evaluations of the objective function defined as the error of classifications, NHU, ILR, and MBS are determined as 9, 0.02, and 170, respectively.

## 4 Results

### 4.1 Experiment one

**FIGURE 6:**
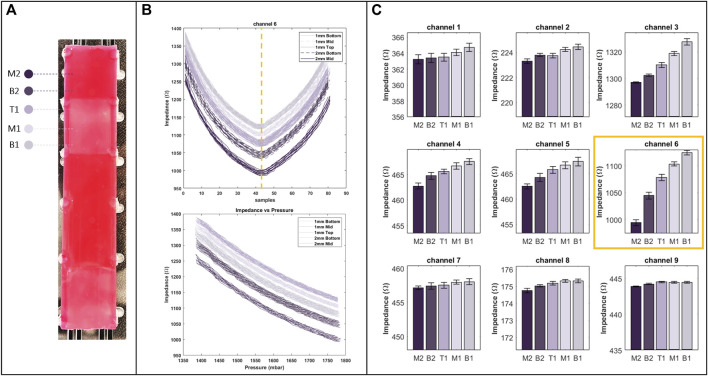
**(A)** Regions with different stiffness on training phantom, used in the first experiment and marked by specific colours as well as labels from M2 to B1 respective to their position on the phantom. **(B)** The raw data of channel six of EIT (top), with respect to pressure (bottom). **(C)** Bar plot of EIT outputs for all of the channels at their peak (indicated by a dashed line on the raw data plot), showcasing the distinction of the outputs (right).

**FIGURE 7:**
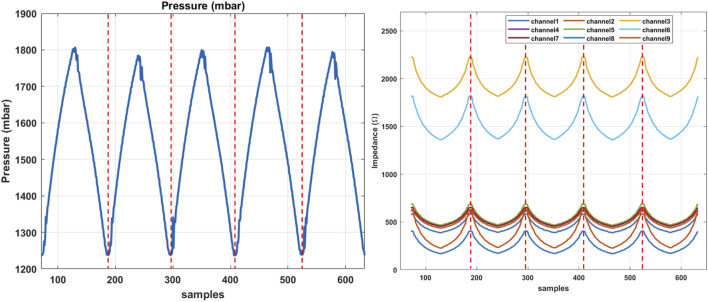
The sequential data obtained from the training phantom in experiment two, indicating the pressure signal on the left and corresponding EIT changes in each channel, sequentially separated by dashed lines.

The results of the first experiments where the distinction of EIT outputs is investigated are shown in Figure 6. The plot in Figure 6B denotes the raw output data for the sixth channel of the EIT protocol. As discussed in Section 3, in the experiment, a trapezoid pressure signal with an amplitude ranging from 1400 to 1700mbar is applied to the manipulator with a high time of 2 s. The raw data illustrate that as the pressure increases, the impedance values decrease correspondingly (see Figure 7B bottom). This behaviour is not specific to the channel noted here and is observed across the entire protocol. The reason for the decline in the impedance value can be described by the increase in the cross-section of the manipulator upon pressurizing the manipulator. As pressure increases, the inflation induces the volume of the conductive fluid, namely, saline, to increase, hence reducing the impedance values according to Pouillet’s law.

Figure 6C illustrates peak EIT channel outputs resulting from interactions with various regions of the tissue phantom. These regions, identified in Figure 6A, are labelled as B1, B2, M1, M2, and T1, representing the bottom of 1 and 2 mm samples, the middle of 1 and 2 mm samples, and the top of 1 mm samples, respectively. The distinction is apparent in nearly all channels, with more pronounced variations in channels measuring impedances at the middle of the manipulator. This corresponds to the middle row in Figure 6C, aligning with electrodes in the middle of the manipulator per the experimental protocol. These high variations result from the middle section experiencing the most significant changes in cross-section. Additionally, the bar plots reveal that the distal section of the manipulator undergoes minimal changes in cross-section.

According to the plots, interaction with the middle of the 2 mm sample yields the lowest impedance value compared to other regions on the phantom. This suggests that cross-section changes are maximized when palpating the middle of the 2 mm sample, indicating the highest resistance in that interaction. This resistance is reflected in increased inflation across different sections of the manipulator. In contrast, interaction with the bottom of the 1 mm sample yields the highest impedance values, implying the least resistance during palpation. The error bars depicted on the plots demonstrate the repeatability of the outputs. Overall, these values demonstrate the acceptable repeatability of EIT outputs.

### 4.2 Experiment two

**FIGURE 8:**
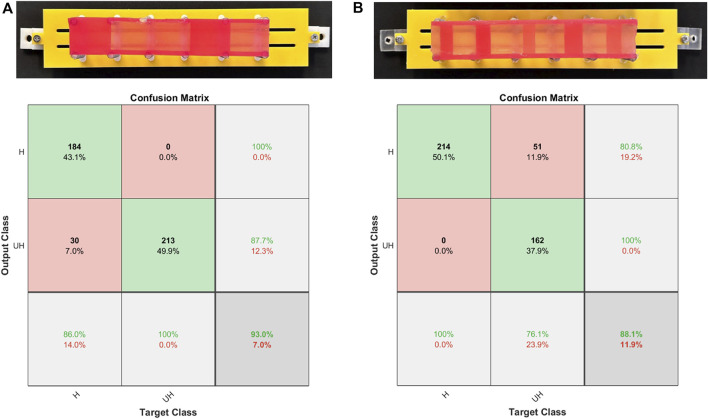
Classification results shown by confusion matrices with respect to the dataset collected on the test phantoms **(A)** and **(B)**. Class H and UH denote the simulated healthy and unhealthy tissues with 1 mm and 2 mm thicknesses, respectively.

A series of sequential data collected during the second experiment is shown in Figure 7, where the pressure signal and corresponding changes in the EIT channels are demonstrated with respect to each channel. Individual sequences are separated by dashed lines. The sampled dataset inputs the LSTM classifier to train the model. The results of the validation using the data sampled from the test phantoms A and B are highlighted in this section. In particular, the data from the test phantoms are not present or involved in the training dataset and the learning algorithm, hence are considered unseen to the classifier. The confusion matrix in Figure 8 demonstrates the classification accuracy of the test dataset from the test phantom A. The phantom incorporates 1 and 2 mm samples, representing healthy and unhealthy tissue samples, respectively. The results indicate that the overall accuracy of the proposed classifier is 93% , implying the correct classification of 397 out of 427 sequences of the input data. The employed algorithms misclassified 30 sequences of healthy target samples as unhealthy tissues, resulting in an overall 7% false positive misclassification. In particular, 30 sequences of the sampled data from 1 mm samples on the phantom A were classified as tissues with high stiffness. In the following paragraphs, the corresponding locations of the misclassified sequences are highlighted using a colour map.

Similarly, Figure 8 demonstrates the validation results of the proposed classifier using the dataset sampled from the tissue phantom B. Contrary to the test phantom A, phantom B incorporates tissue samples with 3 mm and 4 mm thicknesses in addition to 1 and 2 mm samples. Furthermore, the length of the samples is not constant, challenging the classifier more considerably. While the number of tissue samples on phantom A is 5, this number increases to 11 on phantom B. This value is associated with the number of transition regions where the thickness and stiffness of the tissue sample undergo a sudden change. Intuitively, the overall accuracy of the classifications drops to 88.1% , signifying the correct pattern recognition of 374 out of 427 sequential data samples. The remaining data samples are classified as false negative, leading to an error of 11.9% . The confusion matrix illustrates that all of the data samples associated with healthy tissues, represented by 1 mm samples, are recognised correctly by the model. In contrast, the dataset representing the unhealthy tissue samples on phantom A was classified with 100% accuracy. The difference in the fabrication and structure of the phantoms as well as the sampling procedure can contribute to the varying accuracy between the datasets collected from phantoms A and B. In what follows, the respective locations of the misclassified sequences are highlighted using a heat map.

### 4.3 Visualising the location of misclassified data

**FIGURE 9:**
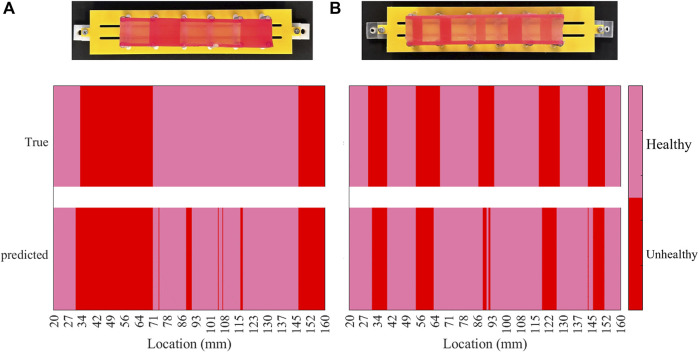
Heat map of the classification results per each phantom, indicating the location of misclassified data samples on the phantoms **(A)** and **(B)**.

Figure 9 visually represents the locations of misclassified sequences on each phantom. Using the fabricated phantom as a reference, the figure illustrates two stiffness classes, visualised as colours with varying brightness. Bright colours represent healthy tissues or 1 mm samples, while darker colours indicate unhealthy tissues, including 2, 3, and 4 mm samples on both phantoms. The heatmap plots are divided into true and predicted visualisations on the vertical axis, denoting ground truth and classifier outputs, respectively. The true part aligns with the fabricated phantom, while the predicted part highlights misclassified palpations. The misclassification distribution on phantom A, as shown in Figure 9, indicates that most misclassifications occur in the middle section. Additionally, some 1 mm samples between 27 and 34 mm are misclassified as unhealthy. Figure 9 demonstrates that misclassified sequences on phantom B are concentrated within transition regions from 1 mm samples to higher stiffnesses. The stiffness changes within these regions are challenging to recognize through EIT outputs or the classifier due to identical stiffness properties. Although classifier accuracy on phantom B is lower due to boundary misclassifications, the centres of high-stiffness tissue samples are clear. However, the misclassified distribution on phantom A complicates the diagnosis by suggesting absent unhealthy tissues. In particular, errors in phantom B are expected at the boundaries between U and UH regions, avoiding false areas for investigation. However, in phantom A, a number of misclassified areas are rather scattered, leading to potential complications such as prolonged operations. To explain the error distribution pattern on phantom A, the anisotropic model of the phantom should be taken into account. The contributing factors include non-uniform moulding of the silicone tissue samples due to uneven distribution of the material during casting, and the relative position of the suspension springs that can vary the stiffness at particular areas. Additionally, since the manipulator traverses the phantoms in constant intervals over the collection of the test data, an asynchronous vertical motion can induce misleading changes in the impedance values. In particular, if the manipulator is lifted while the tip is still contacting the phantom, the manipulator undergoes an unforeseen shift in its shape, impairing the classifier to recognise the input data. The limited response time and Signal-to-Noise Ratio (SNR) of the proposed sensing scheme can amplify the effect of the aforementioned contributing factors.

## 5 Discussion

In this study, we proposed an integrated tactile sensing method within a soft continuum manipulator. The approach holds potential for facilitating *in vivo* tissue palpation during endoscopy, allowing for *in situ* localization and assessment. Experimental investigations conducted on simulated *in vivo* tissue, combined with a machine learning classification method. The distinction between healthy and unhealthy tissue within the phantom is within a 1 mm difference in thickness, which challenges the classifier. The presence of contact resistance at the FPC electrodes contributes to the signal noise. Furthermore, the limited deflections created on the phantoms diminished the differentiability of measurements obtained from distinct regions. This limitation can be addressed by adjusting the stiffness of the manipulator and deploying variable stiffness designs in future works. The results demonstrated the achievement of 93% and 88.1% precision in classifying phantoms with two and four varying stiffness levels, respectively. Notably, previous studies by Yan and Pan (2021) and Xiao et al. (2020) reported accuracy rates of 97% and 99% in anomaly detection using robot-assisted tactile methods. However, these studies employed commercially established sensing modules, partially composed of rigid components and integrable with laparoscopic instruments. In contrast to the soft tactile sensors in the literature Hao et al. (2023); Jones and Damian (2022); Galloway et al. (2019), our proprioceptive tactile system exclusively utilizes the manipulator’s pressurizing fluid, without requiring supplementary attachments on the device’s exterior. This feature preserves the structural stiffness and conserves space for the inclusion of interventional instruments alongside the endoscope.

Apart from the promising results yielded by the proposed algorithm and experimental setup, different aspects of the study require further improvement which will be regarded in future works. Similar to the tactile studies in endoscopy Camboni et al. (2020); Zhao et al. (2023a); Winstone et al. (2016); Venkatayogi et al. (2022); Kara et al. (2023), the fabricated phantom discussed in Section 2 represents the descending colon in a simplistic manner and lacks the corresponding physiological features such as the segmented appearance of the colon or haustra. While the mass-spring phantom possesses a flat surface, the majority of the existing phantoms in the literature consider haustra. However, these phantoms fail to simulate the mechanical properties of the colon such as stiffness, which is the key feature to identify cancerous tissues with palpation. Therefore, designing realistic phantoms that are identical to the large intestine enables accurate and generalized data acquisition with regard to *in vivo* colon tissue palpation Finocchiaro et al. (2023). The promising classification results on unseen tissue samples suggest that the proposed approach holds potential reliability when tested on real tissue in future studies.

The in-plane bending of the manipulator limits its motion to one DOF, inhibiting the validation of the proposed method in bio-realistic 3D phantoms. To enable thorough palpation of the colon and considering the limited motion of the manipulator, a rotational actuation unit is required to complement the vertical motion, complicating the overall delivery system design. In addition to the need for a manipulator capable of 3D movement, enhancing the delivery system to convey the probing manipulator to the targeted tissue is required. Here, a vertical linear motor is leveraged to enable the manipulator traversing the tissue surface. To develop a targeted delivery system and enable palpating tissues further inside the intestine, soft everting robots or alternative everting mechanisms Saxena et al. (2020) can be employed. These systems are capable of compliantly and flexibly conveying the palpating device to the targeted region. Additionally, the palpating manipulator can be mounted on a conventional endoscope delivering the treatment to the surgical site. In this study, controlled motions with random depths were employed; however, the direct implementation of these motions is limited in clinical settings. To generalize the machine learning model in clinical scenarios, it is necessary to incorporate data acquired from previous trials into the training dataset. Furthermore, the deployment of support structures, such as inflatable scaffolds with variable stiffness Runciman et al. (2020), can effectively enlarge the surgical site. Additional anchoring mechanisms, such as double-balloon systems Manfredi et al. (2019), can contribute to the enlargement of the workspace and mitigate external forces. The integration of an additional sensing channel into the soft manipulator can capture readings associated with the random motion of the endoscope, enabling the distinction of tactile measurements. Additionally, the method is also transferable to other endoscopic technologies such as hydraulically-driven everting mechanisms for locomotion in endoscopy Saxena et al. (2020).

The impedance spectroscope records and exports EIT measurements through text files which demands further data post-processing, impairing the real-time classification of the collected data samples. To address the issue, the current EIT system which operates based on TDM and requires constant electrode switching can be upgraded to a Frequency Division Multiplexing (FDM) based acquisition unit Avery et al. (2019). The current setup limits the real-time acquisition and processing of the data samples, induced by the limited sampling rate in TDM. Introducing FDM-based acquisition removes the need to post-process the data, enabling on-demand classification.

In addition to the employment of a TDM-based EIT system, the proposed classifier based on RNN can impose constraints on the real-time classification of the datasets. Although RNN and LSTM algorithms surpass CNN and DNNs in processing temporal data, RNN-based algorithms rely on a recursive mechanism, increasing the training time of the model. Transformer networks predict sequential data following a self-attention approach where the attention and dependency between data points in a sequence are captured more effectively. This procedure is performed by processing the sequential data as a whole rather than point-wise, which is opposed to RNNs Vaswani et al. (2017). However, transformers commonly struggle with overfitting and generalization, introducing data-augmentation techniques as a solution Zeyer et al. (2019). Transformers have demonstrated prominent results in modelling of soft robotics sensory data as in shape reconstruction applications Hu et al. (2023), rendering them promising algorithms to interpret tactile data. Additionally, implementing a model with prediction uncertainty, such as a probabilistic model, could provide insights into the model’s confidence levels regarding its classifications. This approach may offer valuable information about boundaries and potential misdiagnoses, particularly in the middle section of phantom A. In addition to transformers, alternative anomaly detection networks such as Bayesian Online Changepoint Detection (BOCD), One-class Support Vector Machines (SVMs), and Temporal Convolutional Networks (TCNs) will be investigated in future works.

Implementation of data-driven classification methods poses challenges regarding data-collection time. In particular, one of the advantages of elastomer robots in endoscopic applications is their cost-effective manufacturing, offering the development of disposable surgical tools. The disposability of soft robots is achieved by repeatable manufacturing processes allowing fabrication of robots with identical characteristics. However, the conventional fabrication approaches, typically using silicone casting, pose challenges in fabricating repeatable elastomer endoscopes Runciman et al. (2019), requiring larger training datasets to ensure generalization, hence extending data collection time. The issue can be addressed either by devising repeatable manufacturing processes or incorporating augmented data obtained from simulation experiments. Alternatively, the design of bio-realistic phantoms together with devising efficient and time-saving pipelines for data acquisition suggest potential solutions to the matter.

Additional factors influencing classification accuracy encompass the signal-to-noise ratio (SNR) of the EIT hardware system. Enhancements can be achieved by minimising electrode contact resistance. While SNR may seem ineffective when palpated regions are distant (as in the first experiment), close proximity can affect EIT data, complicating classification. In particular, enhanced SNR leads to higher separability of classes of similar stiffnesses. Improvements can be performed by electrode coating techniques using biocompatible and conductive substances Priya Swetha et al. (2018). Furthermore, optimizing the injection-measurement protocol contributes to minimizing the training time and improving the accuracy. The protocol adopted in the study highlights significant shape changes as shown in Figure 6B. The integration of a higher number of electrode pairs in the middle section, optimized through simulations, can reflect the changes in the shape more effectively. Additionally, the material used to fabricate the manipulator can impact the sensitivity of the approach. In particular, softer materials undergo more significant cross-sectional changes upon pressurizing, hence exhibiting more sensitivity when interacting with tissues of varying stiffness.

Future studies will involve fabricating a 3D phantom for enhanced validation of the proposed method in a more realistic context, with subsequent validation on *ex vivo* trials. This prospective phantom will represent additional sections of the large intestine, including the descending colon. To improve manoeuvrability, the proposed palpation method will be applied to a continuum manipulator capable of spatial motion Treratanakulchai et al. (2022). This manipulator, already equipped with surgical instruments, can palpate tissue while triangulating, eliminating the need for an additional palpating instrument. Since the proposed sensing approach allows for scalability, design parameter optimizations will be conducted to make the manipulator’s dimension better aligned with colon anatomical constraints. Further enhancements will focus on achieving real-time classification, incorporating the FDM-EIT hardware system and implementing machine learning models with reduced training time.

## 6 Conclusion

The paper investigates the reliability of classifying tissue samples with varying stiffness by recording shape changes in a soft continuum manipulator driven by hydraulics. The experiments evaluate the application of the proposed approach in classifying tissues of varying stiffness by palpation of a phantom mimicking the colon wall. An in-plane bending manipulator is fabricated, and tissue phantoms with silicone samples of varying thicknesses are designed based on a spring-mass model. Samples with thicknesses exceeding 1 mm simulate early-stage cancerous tissues, considering the stiffness of the mesentery and ligaments in preliminary phantoms. These phantoms undergo palpation by the manipulator, and the collected datasets are used for training and validation by an LSTM classifier.

While a single phantom collects the training dataset, two specific phantoms (A and B) validate the proposed algorithm. Phantom B includes tissue samples with 1, 2, 3, and 4 mm thicknesses, while phantom A only includes 1 and 2 mm thickness samples. The classification method relies on shape changes of the manipulator due to interactions with environments of different stiffness. To track shape changes, EIT sensing and hydraulic pressure values are employed. Sequential data inputs from the corresponding tissue phantoms are used for the classifier. Initially, the separability of EIT data is examined in experiments with a constant pressure signal amplitude. The distinction of EIT measurements from interactions with different tissue phantom regions is evident through the electrodes in the manipulator’s middle. The results of the data-driven classification indicate accurate classification of 93% of data samples from phantom A, dropping to 88.1% for phantom B.

## Data Availability

The raw data supporting the conclusions of this article will be made available by the authors, without undue reservation.

## References

[B1] Abidi H. Gerboni G. Brancadoro M. Fras J. Diodato A. Cianchetti M. (2018). Highly dexterous 2-module soft robot for intra-organ navigation in minimally invasive surgery. Int. J. Med. Robotics Comput. Assisted Surg. 14, e1875. 10.1002/rcs.1875 29205769

[B2] Advincula A. P. Wang K. (2009). Evolving role and current state of robotics in minimally invasive gynecologic surgery. J. Minim. Invasive Gynecol. 16, 291–301. 10.1016/j.jmig.2009.03.003 19423061

[B3] Ahmadi R. Dargahi J. Packirisamy M. Cecere R. (2010). “A new hybrid catheter-tip tactile sensor with relative hardness measuring capability for use in catheter-based heart surgery,” in SENSORS, 2010 (IEEE), 1592–1595.

[B4] Ahmadi R. Packirisamy M. Dargahi J. Cecere R. (2011). Discretely loaded beam-type optical fiber tactile sensor for tissue manipulation and palpation in minimally invasive robotic surgery. IEEE Sensors J. 12, 22–32. 10.1109/jsen.2011.2113394

[B5] Ahn B. Kim Y. Oh C. K. Kim J. (2012). Robotic palpation and mechanical property characterization for abnormal tissue localization. Med. Biol. Eng. Comput. 50, 961–971. 10.1007/s11517-012-0936-2 22772733

[B6] Ahn B. M. Kim J. Ian L. Rha K. H. Kim H. J. (2010). Mechanical property characterization of prostate cancer using a minimally motorized indenter in an *ex vivo* indentation experiment. Urology 76, 1007–1011. 10.1016/j.urology.2010.02.025 20451976

[B7] Alian A. Mylonas G. Avery J. (2023) “Soft continuum actuator tip position and contact force prediction,” in Using electrical impedance tomography and recurrent neural networks, 1–6doi. 10.1109/RoboSoft55895.2023.10121967

[B8] Amadeo T. Lewen D. V. Janke T. Ranzani T. Devaiah A. Upadhyay U. (2022). Soft robotic deployable origami actuators for neurosurgical brain retraction. Front. Robotics AI 8, 731010. 10.3389/frobt.2021.731010 PMC8795889 35096979

[B9] Anju T. Vimala S. (2023). “Finetuned-vgg16 cnn model for tissue classification of colorectal cancer,” in International conference on intelligent sustainable systems (Springer), 73–84.

[B10] Avery J. Runciman M. Darzi A. Mylonas G. P. (2019). “Shape sensing of variable stiffness soft robots using electrical impedance tomography,” in 2019 international conference on robotics and automation (ICRA) (IEEE), 9066–9072.

[B11] Avery J. Shulakova D. Runciman M. Mylonas G. P. Darzi A. (2020). Tactile sensor for minimally invasive surgery using electrical impedance tomography. IEEE Trans. Med. Robotics Bionics 2, 561–564. 10.1109/TMRB.2020.3031636

[B12] Avgousti S. Christoforou E. G. Panayides A. S. Voskarides S. Novales C. Nouaille L. (2016). Medical telerobotic systems: current status and future trends. Biomed. Eng. online 15, 96–44. 10.1186/s12938-016-0217-7 27520552 PMC4983067

[B13] Berthet-Rayne P. Sadati S. M. Petrou G. Patel N. Giannarou S. Leff D. R. (2021). Mammobot: a miniature steerable soft growing robot for early breast cancer detection. IEEE Robotics Automation Lett. 6, 5056–5063. 10.1109/LRA.2021.3068676

[B14] Bhattarai A. Kowalczyk W. Tran T. N. (2021). A literature review on large intestinal hyperelastic constitutive modeling. Clin. Biomech. 88, 105445. 10.1016/j.clinbiomech.2021.105445 34416632

[B15] Bull A. D. (2011). Convergence rates of efficient global optimization algorithms. J. Mach. Learn. Res. 12.

[B16] Camboni D. Massari L. Chiurazzi M. Caliò R. Alcaide J. O. D’Abbraccio J. (2020). Endoscopic tactile capsule for non-polypoid colorectal tumour detection. IEEE Trans. Med. Robotics Bionics 3, 64–73. 10.1109/tmrb.2020.3037255

[B17] Chauhan M. Chandler J. H. Jha A. Subramaniam V. Obstein K. L. Valdastri P. (2021). An origami-based soft robotic actuator for upper gastrointestinal endoscopic applications. Front. Robotics AI 8, 664720. 10.3389/frobt.2021.664720 PMC8141740 34041275

[B18] Chuang C. H. Li T. H. Chou I. C. Teng Y. J. (2016). Piezoelectric tactile sensor for submucosal tumor detection in endoscopy. Sensors Actuators, A Phys. 244, 299–309. 10.1016/j.sna.2016.04.020

[B19] Cianchetti M. Ranzani T. Gerboni G. De Falco I. Laschi C. Menciassi A. (2013). “Stiff-flop surgical manipulator: mechanical design and experimental characterization of the single module,” in 2013 IEEE/RSJ international conference on intelligent robots and systems, 3576–3581. 10.1109/IROS.2013.6696866

[B20] Cianchetti M. Ranzani T. Gerboni G. Nanayakkara T. Althoefer K. Dasgupta P. (2014). Soft robotics technologies to address shortcomings in today’s minimally invasive surgery: the stiff-flop approach. Soft Robot. 1, 122–131. 10.1089/soro.2014.0001

[B21] Ferreira C. A. Melo T. Sousa P. Meyer M. I. Shakibapour E. Costa P. (2018). “Classification of breast cancer histology images through transfer learning using a pre-trained inception resnet v2,” in International conference image analysis and recognition (Springer), 763–770.

[B22] Finocchiaro M. Zabban C. Huan Y. Mazzotta A. D. Schostek S. Casals A. (2023). Physical simulator for colonoscopy: a modular design approach and validation. IEEE Access 11, 36945–36960. 10.1109/ACCESS.2023.3266087

[B23] Fu Z. Jin Z. Zhang C. He Z. Zha Z. Hu C. (2021). The future of endoscopic navigation: a review of advanced endoscopic vision technology. IEEE Access 9, 41144–41167. 10.1109/ACCESS.2021.3065104

[B24] Galloway K. C. Chen Y. Templeton E. Rife B. Godage I. S. Barth E. J. (2019). Fiber optic shape sensing for soft robotics. Soft Robot. 6, 671–684. 10.1089/soro.2018.0131 31241408 PMC6786339

[B25] Gers F. A. Schmidhuber J. Cummins F. (2000). Learning to forget: continual prediction with lstm. Neural Comput. 12, 2451–2471. 10.1162/089976600300015015 11032042

[B26] Gwilliam J. C. Pezzementi Z. Jantho E. Okamura A. M. Hsiao S. (2010). Human vs. robotic tactile sensing: detecting lumps in soft tissue, 21–28.

[B27] Hao J. Zhang Z. Wang S. Shi C. (2023). 2d shape estimation of a pneumatic-driven soft finger with a large bending angle based on learning from two sensing modalities. Adv. Intell. Syst. 5, 2200324. 10.1002/aisy.202370043

[B28] Hu D. Giorgio-Serchi F. Zhang S. Yang Y. (2023). Stretchable e-skin and transformer enable high-resolution morphological reconstruction for soft robots. Nat. Mach. Intell. 5, 261–272. 10.1038/s42256-023-00622-8

[B29] Jones J. Damian D. D. (2022). “A soft fluidic sensor-actuator for active sensing of force and displacement in biomedical applications,” in 2022 IEEE/RSJ international conference on intelligent robots and systems (IROS) (IEEE), 6913–6919.

[B30] Kara O. C. Kim H. Xue J. Mohanraj T. G. Hirata Y. Ikoma N. (2023). “Design and development of a novel soft and inflatable tactile sensing balloon for early diagnosis of colorectal cancer polyps,” in 2023 IEEE/RSJ international conference on intelligent robots and systems (IROS) (IEEE), 10295–10300.

[B31] Kawahara T. Miyata Y. Akayama K. Okajima M. Kaneko M. (2010). Design of noncontact tumor imager for video-assisted thoracic surgery. IEEE/ASME Trans. mechatronics 15, 838–846. 10.1109/tmech.2010.2078830

[B32] Kim U. Kim Y. B. So J. Seok D.-Y. Choi H. R. (2018). Sensorized surgical forceps for robotic-assisted minimally invasive surgery. IEEE Trans. Industrial Electron. 65, 9604–9613. 10.1109/tie.2018.2821626

[B33] Konstantinova J. Jiang A. Althoefer K. Dasgupta P. Nanayakkara T. (2014). Implementation of tactile sensing for palpation in robot-assisted minimally invasive surgery: a review. IEEE Sensors J. 14, 2490–2501. 10.1109/jsen.2014.2325794

[B34] Kwok K. W. Wurdemann H. Arezzo A. Menciassi A. Althoefer K. (2022). Soft robot-assisted minimally invasive surgery and interventions: advances and outlook. Proc. IEEE 110, 871–892. 10.1109/JPROC.2022.3167931

[B35] Lee D.-H. Kim U. Gulrez T. Yoon W. J. Hannaford B. Choi H. R. (2015). A laparoscopic grasping tool with force sensing capability. IEEE/ASME Trans. Mechatronics 21, 1–141. 10.1109/tmech.2015.2442591

[B36] Lopez J. I. Kang I. You W. K. McDonald D. M. Weaver V. M. (2011). *In situ* force mapping of mammary gland transformation. Integr. Biol. 3, 910–921. 10.1039/c1ib00043h PMC3564969 21842067

[B37] Lv C. Wang S. Shi C. (2020). A high-precision and miniature fiber bragg grating-based force sensor for tissue palpation during minimally invasive surgery. Ann. Biomed. Eng. 48, 669–681. 10.1007/s10439-019-02388-w 31686311

[B38] Manfredi L. Capoccia E. Ciuti G. Cuschieri A. (2019). A soft pneumatic inchworm double balloon (spid) for colonoscopy. Sci. Rep. 9, 11109. 10.1038/s41598-019-47320-3 31367005 PMC6668406

[B39] Massalou D. Masson C. Afquir S. Baqué P. Arnoux P.-J. Bège T. (2019). Mechanical effects of load speed on the human colon. J. Biomechanics 91, 102–108. 10.1016/j.jbiomech.2019.05.012 31133391

[B40] Min M. Lehti-Polojärvi M. Hyttinen J. Rist M. Land R. Annus P. (2018). Bioimpedance spectro-tomography system using binary multifrequency excitation. Int. J. Bioelectromagn. 209, 76–79. 10.18154/RWTH-CONV-224930

[B41] Nguyen C. C. Thai M. T. Hoang T. T. Davies J. Phan P. T. Zhu K. (2023). Development of a soft robotic catheter for vascular intervention surgery. Sensors Actuators A Phys. 357, 114380. 10.1016/j.sna.2023.114380

[B42] Nichols K. A. Okamura A. M. (2013). Autonomous robotic palpation: machine learning techniques to identify hard inclusions in soft tissues, , 4384–4389. 10.1109/ICRA.2013.6631198

[B43] Othman W. Lai Z. H. A. Abril C. Barajas-Gamboa J. S. Corcelles R. Kroh M. (2022). Tactile sensing for minimally invasive surgery: conventional methods and potential emerging tactile technologies. Front. Robotics AI 8, 705662. 10.3389/frobt.2021.705662 PMC8777132 35071332

[B44] Polygerinos P. Ataollahi A. Schaeffter T. Razavi R. Seneviratne L. D. Althoefer K. (2010). Mri-compatible intensity-modulated force sensor for cardiac catheterization procedures. IEEE Trans. Biomed. Eng. 58, 721–726. 10.1109/tbme.2010.2095853 21118758

[B45] Polygerinos P. Wang Z. Overvelde J. T. Galloway K. C. Wood R. J. Bertoldi K. (2015). Modeling of soft fiber-reinforced bending actuators. IEEE Trans. Robotics 31, 778–789. 10.1109/TRO.2015.2428504

[B46] Priya Swetha P. D. Manisha H. Sudhakaraprasad K. (2018). Graphene and graphene-based materials in biomedical science. Part. Part. Syst. Charact. 35, 1800105. 10.1002/ppsc.201800105

[B47] Radó J. Dücső C. Földesy P. Szebényi G. Nawrat Z. Rohr K. (2018). 3d force sensors for laparoscopic surgery tool. Microsyst. Technol. 24, 519–525. 10.1007/s00542-017-3443-4

[B48] Rateni G. Cianchetti M. Ciuti G. Menciassi A. Laschi C. (2015). Design and development of a soft robotic gripper for manipulation in minimally invasive surgery: a proof of concept. Meccanica 50, 2855–2863. 10.1007/s11012-015-0261-6

[B49] Runciman M. Avery J. Zhao M. Darzi A. Mylonas G. P. (2020). Deployable, variable stiffness, cable driven robot for minimally invasive surgery. Front. Robotics AI 6, 141. 10.3389/frobt.2019.00141 PMC7805644 33501156

[B50] Runciman M. Darzi A. Mylonas G. P. (2019). Soft robotics in minimally invasive surgery. Soft Robot. 6, 423–443. 10.1089/soro.2018.0136 30920355 PMC6690729

[B51] Sarwinda D. Paradisa R. H. Bustamam A. Anggia P. (2021). Deep learning in image classification using residual network (resnet) variants for detection of colorectal cancer. Procedia Comput. Sci. 179, 423–431. 10.1016/j.procs.2021.01.025

[B52] Saxena A. Pauli E. M. Haluck R. S. Fell B. Moore J. (2020). Tubular locomotion and positioning using tip eversion for endoscopy. J. Med. Devices 14, 021004. 10.1115/1.4046433

[B53] Scimeca L. Hughes J. Maiolino P. He L. Nanayakkara T. Iida F. (2022). Action augmentation of tactile perception for soft-body palpation. Soft Robot. 9, 280–292. 10.1089/soro.2020.0129 34432994 PMC9347261

[B54] Sornkarn N. Nanayakkara T. (2017). Can a soft robotic probe use stiffness control like a human finger to improve efficacy of haptic perception? IEEE Trans. Haptics 10, 183–195. 10.1109/TOH.2016.2615924 27775537

[B55] Stella F. Hughes J. Rus D. Santina C. D. (2023). Prescribing cartesian stiffness of soft robots by co-optimization of shape and segment-level stiffness. Soft Robot. 10, 701–712. 10.1089/soro.2022.0025 37130308

[B56] Stewart D. C. Berrie D. Li J. Liu X. Rickerson C. Mkoji D. (2018). Quantitative assessment of intestinal stiffness and associations with fibrosis in human inflammatory bowel disease. PLoS ONE 13, e0200377. 10.1371/journal.pone.0200377 29995938 PMC6040714

[B57] Stilli A. Wurdemann H. A. Althoefer K. (2014). “Shrinkable, stiffness-controllable soft manipulator based on a bio-inspired antagonistic actuation principle,” in 2014 IEEE/RSJ international conference on intelligent robots and systems, 2476–2481. 10.1109/IROS.2014.6942899

[B58] Tanaka Y. Doumoto K. Sano A. Fujimoto H. (2010). “Development of a sensor system with syringe based on tactile sensing using balloon expansion,” in 2010 IEEE international conference on robotics and automation, 4861–4866. 10.1109/ROBOT.2010.5509877

[B59] Tang Y. Liu H. Pan J. Zhang Z. Xu Y. Yao N. (2021). Optical micro/nanofiber-enabled compact tactile sensor for hardness discrimination. ACS Appl. Mater. Interfaces 13, 4560–4566. 10.1021/acsami.0c20392 33435667

[B60] Tolley M. T. Shepherd R. F. Mosadegh B. Galloway K. C. Wehner M. Karpelson M. (2014). A resilient, untethered soft robot. Soft Robot. 1, 213–223. 10.1089/soro.2014.0008

[B61] Treratanakulchai S. Franco E. Garriga-Casanovas A. Minghao H. Kassanos P. y Baena F. R. (2022). “Development of a 6 dof soft robotic manipulator with integrated sensing skin,” in 2022 IEEE/RSJ international conference on intelligent robots and systems (IROS) (IEEE), 6944–6951.

[B62] Trueeb C. Sferrazza C. D’Andrea R. (2020). “Towards vision-based robotic skins: a data-driven, multi-camera tactile sensor,” in 2020 3rd IEEE international conference on soft robotics (RoboSoft) (IEEE), 333–338.

[B63] Van Lewen D. Janke T. Lee H. Austin R. Billatos E. Russo S. (2023). A millimeter-scale soft robot for tissue biopsy procedures. Adv. Intell. Syst. 5, 2200326. 10.1002/aisy.202200326 37637939 PMC10456987

[B64] Vaswani A. Shazeer N. Parmar N. Uszkoreit J. Jones L. Gomez A. N. (2017). Attention is all you need. Adv. neural Inf. Process. Syst. 30.

[B65] Venkatayogi N. Kara O. C. Bonyun J. Ikoma N. Alambeigi F. (2022). “Classification of colorectal cancer polyps via transfer learning and vision-based tactile sensing,” in 2022 IEEE sensors (IEEE), 1–4.

[B66] Wang Q. Chen E. Cai Y. Chen C. Jin W. Zheng Z. (2016). Preoperative endoscopic localization of colorectal cancer and tracing lymph nodes by using carbon nanoparticles in laparoscopy. World J. Surg. Oncol. 14, 231. 10.1186/s12957-016-0987-1 27577559 PMC5004270

[B67] Wanninayake I. B. Seneviratne L. D. Althoefer K. (2012). Novel indentation depth measuring system for stiffness characterization in soft tissue palpation, , 4648–4653. 10.1109/ICRA.2012.6225127

[B68] Williamson T. Song S.-E. (2022). “Robotic surgery techniques to improve traditional laparoscopy,” in JSLS: journal of the society of laparoscopic and robotic surgeons 26. 10.4293/JSLS.2022.00002 PMC9135605 35655469

[B69] Winstone B. Melhuish C. Pipe T. Callaway M. Dogramadzi S. (2016). Toward bio-inspired tactile sensing capsule endoscopy for detection of submucosal tumors. IEEE Sensors J. 17, 848–857. 10.1109/jsen.2016.2627798

[B70] Xiao B. Xu W. Guo J. Lam H. K. Jia G. Hong W. (2020). Depth estimation of hard inclusions in soft tissue by autonomous robotic palpation using deep recurrent neural network. IEEE Trans. Automation Sci. Eng. 17, 1791–1799. 10.1109/TASE.2020.2978881

[B71] Yan Y. Pan J. (2021). Fast localization and segmentation of tissue abnormalities by autonomous robotic palpation. IEEE Robotics Automation Lett. 6, 1707–1714. 10.1109/LRA.2021.3058870

[B72] Yu Y. Si X. Hu C. Zhang J. (2019). A review of recurrent neural networks: lstm cells and network architectures. Neural Comput. 31, 1235–1270. 10.1162/neco_a_01199 31113301

[B73] Zeyer A. Bahar P. Irie K. Schlüter R. Ney H. (2019). “A comparison of transformer and lstm encoder decoder models for asr,” in 2019 IEEE automatic speech recognition and understanding workshop (ASRU) (IEEE), 8–15.

[B74] Zhang B. Fan Y. Yang P. Cao T. Liao H. (2019). Worm-like soft robot for complicated tubular environments. Soft Robot. 6, 399–413. 10.1089/soro.2018.0088 31180823

[B75] Zhang Qiu P. Tan Y. Thompson O. Cobley B. Nanayakkara T. (2022). Soft tissue characterisation using a novel robotic medical percussion device with acoustic analysis and neural networks. IEEE Robotics Automation Lett. 7, 11314–11321. 10.1109/LRA.2022.3191053

[B76] Zhao S. Nguyen C. C. Hoang T. T. Do T. N. Phan H.-P. (2023a). Transparent pneumatic tactile sensors for soft biomedical robotics. Sensors 23, 5671. 10.3390/s23125671 37420836 PMC10302895

[B77] Zhao S. Nguyen C. C. Hoang T. T. Do T. N. Phan H. P. (2023b). Transparent pneumatic tactile sensors for soft biomedical robotics. Sensors 23, 5671. 10.3390/s23125671 37420836 PMC10302895

